# Alginate Conjugation Increases Toughness in Auricular Chondrocyte Seeded Collagen Hydrogels

**DOI:** 10.3390/bioengineering10091037

**Published:** 2023-09-04

**Authors:** Leigh Slyker, Lawrence J. Bonassar

**Affiliations:** 1 Meinig of Biomedical Engineering, Cornell University, Ithaca, NY 14853, USA; 2 Sibley School of Mechanical and Aerospace Engineering, Cornell University, Ithaca, NY 14853, USA

**Keywords:** collagen, functionalization, complexation, extensibility, toughness

## Abstract

Current auricular cartilage replacements for pediatric microtia fail to address the need for long-term integration and neocartilage formation. While collagen hydrogels have been successful in fostering neocartilage formation, the toughness and extensibility of these materials do not match that of native tissue. This study used the N-terminal functionalization of collagen with alginate oligomers to improve toughness and extensibility through metal–ion complexation. Alginate conjugation was confirmed via FTIR spectroscopy. The retention of native collagen fibrillar structure, thermal gelation, and helical conformation in functionalized gels was confirmed via scanning electron microscopy, oscillatory shear rheology, and circular dichroism spectroscopy, respectively. Alginate–calcium complexation enabled a more than two-fold increase in modulus and work density in functionalized collagen with the addition of 50 mM CaCl_2_, whereas unmodified collagen decreased in both modulus and work density with increasing calcium concentration. Additionally, the extensibility of alginate-functionalized collagen was increased at 25 and 50 mM CaCl_2_. Following 2-week culture with auricular chondrocytes, alginate-functionalization had no effect on the cytocompatibility of collagen gels, with no effects on cell density, and increased glycosaminoglycan deposition. Custom MATLAB video analysis was then used to quantify fracture toughness, which was more than 5-fold higher following culture in functionalized collagen and almost three-fold higher in unmodified collagen.

## 1. Introduction

Deformity of the auricle (outer ear) as a result of the congenital disorder microtia affects 1 in 7000–8000 children, with higher prevalence in Hispanic, Asian, and Native American populations and in males more generally [1]. Psychological morbidities associated with the condition have been well documented [1,2], and auricle deformity has also been implicated in sound lateralization [3]. However, while current treatments for microtia are effective in reducing psychological morbidities, such treatments are surgically intensive, and can take years to complete [1]. Thus, for children affected with microtia, these time-intensive procedures fail to completely mitigate the harmful effects of auricle defects.

The clinical standard for auricle reconstruction is autologous costal cartilage grafting, with 88% of surgeons reporting it as their preferred method [4]. Autologous graft reconstruction is primarily dependent on successful cartilage harvest, which can be a barrier to surgery for young patients, who have insufficient cartilage volume for effective reconstruction. Even in older patients, however, there is incidence of complications such as chest wall deformity, pneumothorax (lung collapse), and pain at the harvest site. As such, the development of biomaterials for auricle replacement has the potential to mitigate the psychological effects of deformations in school-age children, which have been shown to increase with age [4].

Alloplastic auricle replacements have been developed to mitigate the complications associated with autologous costal cartilage grafts. However, while the current generation of porous polyethylene (MEDPOR^®^) replacements have been successful in reducing the incidence of extrusion and infection to 5–10%, [5,6] these implants are not biodegradable and cannot facilitate neocartilage formation. Thus, various biopolymer hydrogel materials have been developed to foster extracellular matrix deposition and remodeling by auricular chondrocytes in situ [7,8,9,10,11,12,13,14]. These biopolymer hydrogels, while necessary for cytocompatibility, lack the toughness and extensibility needed to match native auricular cartilage. As such, these materials are generally protected by various shell or cage materials to bolster their mechanical properties [15]. These multi-material strategies present additional complications in processing and clinical implementation, motivating the development of biopolymer hydrogels with toughness and extensibility comparable to native auricular cartilage.

High-density collagen hydrogels, used in concert with auricular chondrocytes, have demonstrated neo-cartilage formation after in vivo development [16,17,18]. The deposition of collagen and glycosaminoglycans (GAGs) led to stiffness on par with native auricular cartilage. However, elastin deposition was limited even after 3 months of development in murine subcutaneous implantation [16,17,18]. As such, the extensibility and toughness of tissue-engineered (TE) cartilage is not expected to match that of native auricular cartilage, particularly at early times after implantation [16,17,18]. Additionally, the collagen hydrogels used in TE cartilage have a tensile modulus of 5–15 kPa [19], which is significantly lower than the current clinical standards of costal cartilage (1–20 MPa) [20] or porous polyethylene (150–250 MPa) [21,22]. As such, the development of more extensible and tougher hydrogels would be beneficial to auricular tissue engineering efforts.

Mussel byssal threads have been highlighted as a material with exceptional extensibility and toughness, with minimal damage at tensile strains up to 70% [23,24] and failure strains over 100% [25]. The main biochemical mechanism behind the performance of mussel byssus collagen is the presence of metal–catecholate complexes formed through the coordination of DOPA (l-3,4-dihydroxyphenylalanine) residues, which are formed through the post-transcriptional modification of tyrosine residues, and metals such as iron (Fe^2,3+^) [24]. These metal–ion complexes absorb energy upon extension through ion bond breakage. Then, upon relaxation, the complexes reform, leading to exceptional performance over repeated loading cycles.

This mechanism has been applied through the complexation of alginate with calcium ions (Ca^2+^). Through the incorporation of alginate and similar polysaccharides into hydrogels, several groups have demonstrated the ability of alginate–calcium complexes to significantly improve the fracture energy, extensibility, and self-healing of hydrogel materials [26]. In these materials, deformation at the leading edge of cracks breaks apart calcium–alginate complexes, absorbing energy and reducing stress concentration immediately ahead of the crack edge. Due to the reversibility of calcium–alginate complex formation, these materials also have self-healing behavior, so that losses of modulus and work density following plastic deformation are substantially recovered over time. Still, these materials take advantage of highly crosslinked synthetic polymers with minimal biodegradability or biocompatibility, limiting their application to tissue engineering applications.

Despite the successful application of these metal–ion complex strategies in other hydrogels, [27,28,29] their application to collagen biomaterials has not been explored. Several groups have combined collagen with complex-forming materials such as alginate [30,31,32] and chitosan [33]. However, while successful in modulating the mechanical properties and response of cells seeded on these materials, these materials do not form robust networks of collagen fibrils due either to phase separation, interference with collagen fibrillogenesis, or as the result of fabrication conditions. The covalent modification of collagen through various crosslinking strategies has likewise affected the gelation and fibril structure of the resultant materials. This interference with gelation and fibrillogenesis can be ascribed to multiple factors, including the denaturation of the main helical region of collagen, interference in the intermolecular interactions between adjacent collagen molecules within each fibril, and the disruption of telopeptide–telopeptide interactions. The role of telopeptides in the fibrillogenesis of collagen is especially relevant in covalent modification strategies, as many of the reactive species present on collagen molecules are more prevalent in the telopeptide domain rather than the main helical region.

Due to the adverse effects of helical region and telopeptide conjugation strategies, N-terminal specific reactions have the potential for functionalization with the preservation of collagen fibrillogenesis and thermal gelation. Thus, this study evaluates the use of N-terminal-specific functionalization of collagen with alginate oligomers to improve the toughness and extensibility of auricular biomaterials. Fourier-transform IR spectroscopy was used to determine the extent of functionalization. The fibrillar network nanostructure, structural stability, and gelation time of alginate-functionalized collagen (ColAlg) and native collagen (Col) was evaluated with scanning electron microscopy and oscillatory shear rheology, respectively. The maintenance of the main chain helical structure was likewise evaluated using circular dichroism spectroscopy. Following confirmation of a robust fibril and network structure, the tensile modulus and extensibility were evaluated with notched tensile testing, as a function of calcium concentration. Finally, cytocompatibility was assessed using seeding gels with auricular chondrocytes and evaluating the structural, biochemical, and mechanical development of both functionalized and native collagen. Specifically, the increase in fracture toughness as a result of seeding was quantified through a custom MATLAB video tracking protocol which represents a significant advancement in the accessibility of fracture toughness evaluation techniques.

## 2. Materials and Methods

### 2.1. Preparation of Stock Collagen Solutions

Type I collagen was extracted from rat tail tendons as previously described [34]. Briefly, rat tail tendons (BioIVT, Westbury, NY, USA) were solubilized in 0.1% (*v*/*v*) acetic acid (Sigma, St. Louis, MO, USA) at a concentration of 6.67 g/L at 4 °C for a minimum of 48 h. Solutions were centrifuged at 9000× rpm (relative centrifugal force of 5625) for 90 min at 4 °C with lyophilization of the supernatant to yield a collagen sponge. The lyophilized collagen was then reconstituted in 0.1% acetic acid at a concentration of 15 mg/mL.

### 2.2. Alginate Conjugation

Oligomeric alginate was synthesized through the acid-catalyzed hydrolysis [35] of PRONOVA UP LVG Alginate (Novamatrix, Sandvika, Norway), with pre-hydrolysis at pH 5.6 for 3.5 h, followed by hydrolysis at pH 3.5 for 16 h under nitrogen at 95 °C, neutralization, and lyophilization to yield an average molecular weight of 3 kDa as determined via rheology. Briefly, alginate samples were tested at 5, 10, and 25 mg/mL in 0.1 M NaCl over shear rates of 1–10 s^−1^ at 25 °C on a TA Instruments DHR3 with a 40 mm 2° cone-on-plate geometry. Molecular weight was then calculated using the Mark–Houwink–Sakurada equation with constants from Martinsen et al. [36]. A plot of molecular weight vs. hydrolysis time has been included for reference (Supplementary Figure S1).

**Figure 1:**
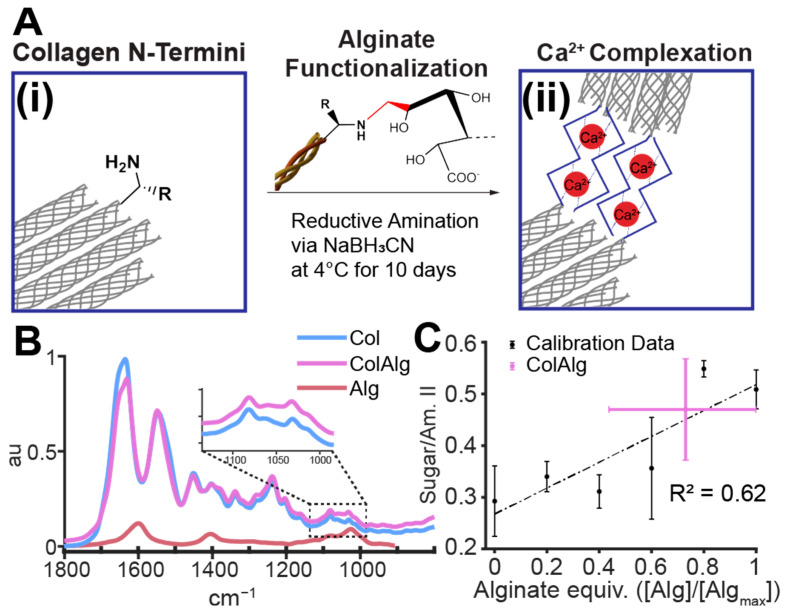
Reaction scheme (**A**) for alginate functionalization of collagen n-termini (**i**) to enable calcium complex formation (**ii**). FTIR spectra of alginate-functionalized and unmodified collagen, alginate stock (**B**), and sugar/amide II peak area calibration to determine extent of functionalization (**C**). *n* = 6 for all spectra and samples.

Oligomeric alginate was then conjugated to the N-terminal amine of collagen through sodium cyanoborohydride-catalyzed reductive alkylation [37]. Alginate and collagen were each dissolved in 0.1% acetic acid at 5 and 15 mg/mL, respectively. Sodium cyanoborohydride (Alfa Aesar, Tewksbury, MA, USA) was dissolved in 0.1% acetic acid under N_2_ protection and added to a solution of alginate, sodium chloride, and acetic acid at 4 °C. The collagen solution was then added to the reaction mixture with gentle mixing to form a one-pot reaction with 9 mg/mL collagen, 0.25 mg/mL alginate, 7.89 mg/L sodium cyanoborohydride, and 300 mOsm NaCl to prevent collagen–alginate precipitation (Figure 1A). The pH of each reaction mixture was confirmed to be approximately 3.5. Additionally, the solubility of both collagen and alginate oligomers in 0.1% acetic acid was confirmed visually prior to reaction and is well supported by prior literature [31,38].

The above mixtures were reacted under gentle mixing at 4 °C for 5, 10, and 15 days to optimize conjugation. The conjugation fraction was found to plateau at 10 days, with increases in the gelation time of 15-day collagen–alginate. Thus, 10-day reaction mixtures were used moving forward. All mixtures were dialyzed against 0.05 M acetic acid at 4 °C in 10 kDa MWCO tubing (Spectra/Por^®^) for 3–4 days with 9 total changes to remove unreacted alginate, cyanoborohydride, and sodium chloride. Resultant collagen–alginate conjugates (ColAlg) and reaction controls (Col), which were processed identically to ColAlg but without alginate, were then lyophilized and resuspended in 0.1% acetic acid at 15 mg/mL (Figure 1B).

### 2.3. Fourier-Transform IR Spectroscopy

FTIR spectra were collected for stock collagen, ColAlg, and collagen–alginate mixtures to determine the extent of conjugation achieved via reductive amination. For calibration mixtures, alginate was added to collagen solutions at 5 intervals up to molar equivalency with collagen at 9 mg/mL, using a molecular weight of 115 kDa for each collagen chain. Each mixture was then lyophilized and analyzed using the attenuated total reflectance detector on a Bruker Vertex 80V Vacuum FTIR Spectrometer (Billerica, MA, USA). Spectra were collected from 2000 to 500 cm^−1^ at 5 cm^−1^ resolution and 6 scans per wavenumber. A total of 6 samples were analyzed per group, with the averages of normalized spectra used in all calculations. A custom MATLAB (version R2023a, MathWorks, Natick, MA, USA) code was used for all spectral plotting and analysis.

### 2.4. Scanning Electron Microscopy

Hydrogels were prepared from each collagen source by means of neutralization using a solution of 1 M sodium hydroxide (Avantor, Center Valley, PA, USA), HEPES buffer (Corning Life Sciences, Oneonta, NY, USA), and de-ionized (DI) water to a final collagen concentration of 8 mg/mL and osmolarity of 300 mOsm/kg H_2_O, as previously described [39]. All solutions were kept on ice before mixing and neutralized to a pH of 7.5 immediately prior to testing.

Gels were deposited in the wells of 24-well plates (Corning Life Sciences, Oneonta, NY, USA) and incubated at 37 °C for 2 h. Following incubation, gels were fixed in 4% formalin in 1X PBS (Fisher Scientific, Fairlawn, NJ, USA) at room temperature for 1 h. Subsequently, gels were rinsed three times with 1× PBS (Corning Life Sciences, Oneonta, NY, USA) for 10 min and then rinsed twice with DI water for 10 min. Gels were then fixed in 1% osmium tetroxide (OsO_4_) (Alfa Aesar, Ward Hill, MA, USA) in DI water for 1 h. Samples were then dried with a series dilution of water into ethanol (30%, 50%, 70%, 90%, then 100% (×2) ethanol in water) for 10 min each. After soaking in ethanol for 1 day, samples were dried via critical point CO_2_ for 1 day or until complete ethanol removal.

Directly prior to imaging, samples were fixed to 18 mm aluminum specimen mounts with double-sided copper tape and sputter-coated with gold/palladium alloy for 15 s at a target current of 20 mA. Samples were then imaged on a Zeiss GeminiSEM 500 (White Plains, NY, USA) field emission SEM. Samples were prepared and imaged in triplicate.

### 2.5. Oscillatory Shear Rheology

To determine the stability of gels formed from alginate-functionalized collagen, as well as the speed of that gelation, rheology was performed on a TA Instruments DHR3 rheometer using a 25 mm parallel plate geometry and a gap height of 1 mm. Sinusoidal oscillation was then applied at 0.1 Hz and 0.5% strain for 5 min at 4 °C followed by 15 min at 37 °C, with the storage and loss modulus recorded at 0.1 Hz. All tests were performed with polyethyleneimine/glutaraldehyde-treated cover slips, as previously described, [40] to ensure no-slip boundary conditions. The final storage modulus (G′) was calculated as the average storage modulus in the last 300 s of testing. Gelation time (t_g_) was calculated as the point of maximum change in storage modulus with time. A custom MATLAB code was used for all rheological data analysis.

### 2.6. Circular Dichroism Spectroscopy

To assess the helical structure of alginate-functionalized collagen, collagen solutions were diluted to 0.01 mg/mL in 1× PBS to form stable suspensions. Suspensions were then measured in triplicate in 10 mm-path-length quartz cuvettes (Thor Labs, Newton, NJ, USA). Circular dichroism spectra were measured on a Jasco J-1500 Circular Dichrosim Spectrophotometer (Easton, MD, USA) at 4 °C at a detection scale of 200 mdeg/0.1 dOD from 190–260 nm at a resolution of 0.5 nm. Spectra were scaled to molar ellipticity (θ) using a mean residue weight of 91 Da [41].

### 2.7. Tensile Mechanics

Acellular tensile mechanics and fracture sensitivity were determined through notched tensile testing, as previously described [42]. Briefly, samples (prepared as described above) were deposited in 7.5 × 50 × 3 mm PDMS molds, incubated at 37 °C for 2 h, and tested after 24 h post-curing at 4 °C. Immediately prior to testing, samples were split in half lengthwise to yield samples with 25 mm length and loaded into the tensile grips of a TA Instruments ElectroForce 5500 (New Castle, DE, USA) with a gauge length of 10–15 mm. Cell-seeded samples were likewise trisected to yield ~15 × 5 × 2 mm sections, which were likewise loaded with a gauge length of ~10 mm. Samples were then pre-fractured by one third of their width using a razor blade and pulled in tension at 10 mm/min to a final displacement of 10 mm. Crack propagation was recorded using a SONY α3000 fitted with an F3.5–5.6 OSS zoom lens at 30 frames per second.

All mechanical properties were determined using a custom MATLAB function (Supplementary Information). The modulus was determined through fitting the linear elastic region of each sample’s stress–strain curve. The strain at ultimate tensile strength (UTS) was determined as the strain at the maximum stress recorded for each sample. Work density was calculated as the area under the stress–strain curve for each sample. Fracture toughness was calculated using a custom crack edge tracking software (Supplementary Information). Using an analytical framework published by Tutwiler et al. [43] and further validated by Darkes-Burkey et al. [42], decreases in stiffness in force–extension plots were referenced to increases in crack tip extension, with the calculation of fracture toughness (Γ) as

(1) $$\Gamma \approx -\frac{1}{2t}\Delta_{c}^{*2}\left(\frac{d\left(\frac{F}{\Delta }\right)}{dc}\right)|c=c^{*}$$

where t is the sample thickness, $c^{*}$ and $\Delta_{c}^{*}$ are the crack extension and sample displacement, respectively, and $\frac{F}{\Delta }$ is the sample stiffness. Statistical analysis as described below was performed on the calculated mechanical properties of each sample.

### 2.8. Auricular Chondrocyte Seeding

Auricular chondrocytes were isolated from neonatal bovine ears as previously described [44] and seeded in gels at 25 × 10^6^ mL^−1^ (30 × 10^6^ cells in each 1.2 mL gel) and incubated for 45 min at 37 °C in custom 2 × 2 × 0.3 mm PDMS molds to form gels. Chondrocyte-seeded gels were then cultured in DMEM with 4500 μg/mL glucose, 10% FBS, and 1% Antibiotic/Antimicotic solution (Corning Life Sciences, Oneonta, NY, USA) for 2 weeks. Construct contraction (A/A_0_) following culture was determined via gross image analysis in Fiji [44].

Separately, chondrocyte-seeded gels were analyzed for viability using a LIVE/DEAD Viability/Cytotoxicity Kit for Mammalian Cells (Thermo Fisher Scientific, Waltham, MA, USA) with calcein AM and ethidium homodimer-1 concentrations of 1 μL per mL of PBS. Following 30 min of staining and 5 min of de-staining in fresh PBS, gels were imaged directly using a 10× objective mounted on a Zeiss LSM 880 inverted confocal microscope. The quantification of cell viability was performed using a MATLAB cell counting package [45].

### 2.9. Biochemical Analysis

Following tensile analysis, samples were collected from between the test frame grips and weighed before and after lyophilization for 48 h to obtain wet and dry weights. Dried samples were then digested with papain phosphate-buffered saline with 10 mM ethylenediaminetetraacetic acid for 18 h. Following digestion, deoxyribonucleic acid (DNA) and hydroxyproline (Hypro) content was determined via previously established assays using Hoescht and 4-(dimethylamino)benzaldehyde, respectively [46,47]. Glycosaminoglycan (GAG) content was determined using dimethyl-methylene blue at a pH of 1.5 to avoid alginate–dye conjugation, which can occur at higher pH levels [48].

### 2.10. Histology

Following tensile analysis, samples were fixed in 4% formalin in 1× PBS (Fisher Scientific, Fairlawn, NJ, USA) and then dehydrated in 70% ethanol for 24 h. Samples were then embedded in paraffin blocks, and sectioned, by the Animal Health Diagnostic Center (College of Veterinary Medicine, Cornell University, Ithaca, NY, USA). Samples were then stained with Alcian Blue and Fast Red counterstain. Images were then captured on a Nikon Eclipse TE2000-S microscope (Nikon Instruments, Melville, NY, USA) with a SPOT RT camera (Diagnostic Instruments, Sterling Heights, MI, USA).

### 2.11. Statistical Analysis

Rheological performance was compared using Student’s *t*-test (α = 0.05). Tensile mechanic contraction and biochemical development were compared using 1-way or 2-way (on group, culture conditions) ANOVA with Tukey’s HSD post hoc analysis (α = 0.05) using RStudio Version: 2023.06.1+524 (Posit Software, Boston, MA, USA) running R version 4.3.1.

## 3. Results

To assess the extent of the reaction achieved with the cyanoborohydride-catalyzed reductive amination between alginate and collagen n-termini, the FTIR spectrum of ColAlg was compared to that of unmodified collagen. With component peaks of 1600, 1400, 1080, and 1030 cm^−1^ (Figure 1B), alginate conjugation resulted in increased peak area in the 990 to 1135 cm^−1^ region of the collagen spectra, which is generally attributed to native collagen glycation (Figure 1B, inset). In order to determine the degree of conjugation achieved in the conjugation reaction, we compared the sugar-to-amide II ratio of ColAlg to those of collagen alginate mixtures equivalent to 0 to 100% conjugation to make a calibration curve ($R^{2}=0.62$). Next, the sugar-to-amide II ratio of alginate-functionlized collagen was compared to that of standards, resulting in a calculated conjugation efficiency of 0.73 ± 0.29 (Figure 1C).

**Figure 2:**
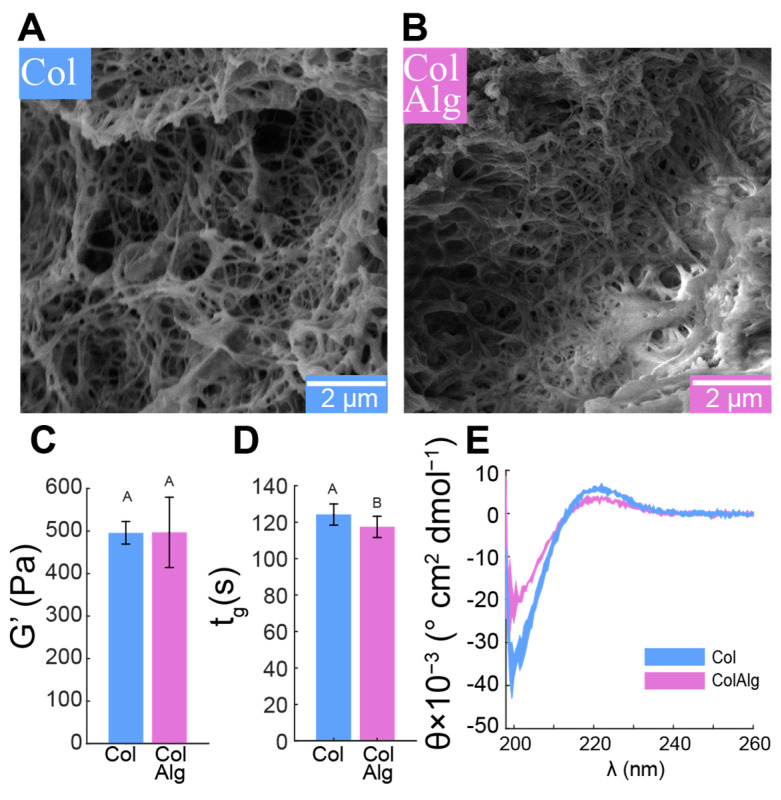
SEM micrographs of collagen (**A**) and ColAlg (**B**). Storage modulus (**C**), gelation time (**D**), and circular dichroism spectra (**E**) of Col and ColAlg. *n* = 3 for all samples. Shared letters denote no significant difference.

To assess the effect of alginate functionalization on fibrillogenesis, we used freeze–fracture scanning electron microscopy to directly image the gel fibrillar structure, confirmed the stability and kinetics of fibril network formation with oscillatory shear rheology, and finally confirmed normal helical conformation using circular dichroism spectroscopy. Fibrils with smooth, randomly distributed morphology and a diameter of 89 ± 22 nm were found in both the Col and ColAlg samples (Figure 2A,B). Consistent with the formation of a functionally robust fibril network in both gels, the shear modulus of Col and ColAlg was 496 ± 61 Pa overall (Figure 2C), which is normal for gels at this concentration. Similarly, the gelation time of each gel was (121 ± 6 s) upon heating from 4 to 37 °C (Figure 2D), which is again normal for collagen gels, indicating that the kinetics of fibrillogenesis in functionalization collagen were not negatively affected by alginate conjugation. As a final confirmation of normal helical formation in both Col and ColAlg, circular dichroism spectroscopy confirmed characteristic spectra indicative of normal collagen structure, with a negative and positive peak at 190 nm and 220 nm, respectively (Figure 2E).

**Figure 3:**
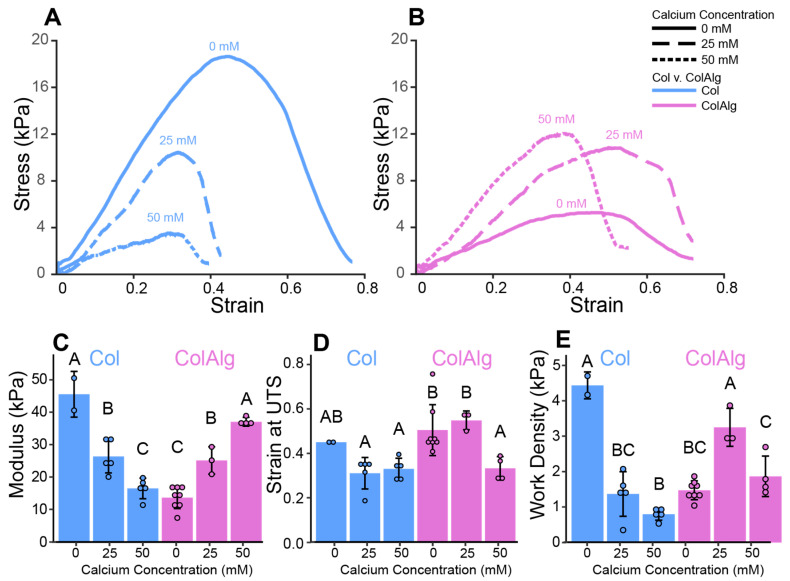
Representative stress–strain curves for Col (**A**) and ColAlg (**B**) gels, with calculated moduli (**C**), strain at ultimate tensile stress (**D**), and work density (**E**). *n* = 2–8. Shared letters denote no significant difference.

Once the fibril-scale structure and stability were confirmed, notched tensile testing was used to determine the mechanical properties of each material as a function of the concentration of calcium, which is expected to enable calcium–alginate complexes in ColAlg. Specifically, the modulus, strain at UTS, and work density were used to evaluate linear elasticity, failure properties, and the total energy of deformation, respectively. Crack edge tracking of notched tension videos was then used to calculate fracture toughness for each material. Comparisons between materials and conditions were then performed between the calculated modulus, strain at UTS, and work density values. Both the modulus and work density of unmodified Col gels decreased, with 40 and 60 percent decreases in modulus (*p* = 1.0 × 10^−4^, *p* = 2.8 × 10^−8^) (Figure 3A,C), and 70 and 80 percent decreases in work density (*p* = 1.1 × 10^−7^, 1.5 × 10^−9^) (Figure 3A,E) with the addition of 25 and 50 mM CaCl_2_, respectively. The strain at UTS trended towards decreased extensibility with calcium addition (*p* = 0.32) (Figure 3D). Modified ColAlg gels, however, increased in modulus 1.8- and 2.7-fold (*p* = 3.0 × 10^−3^, 7.6 × 10^−9^) (Figure 3B,C) with the addition of 25 and 50 mM CaCl_2_, respectively. Work density also increased 2.2-fold (*p* = 8.1 × 10^−5^) (Figure 3B,E) with the addition of 25 mM CaCl_2_. Extensibility at 50 mM CaCl_2_ was 30 and 40 percent lower than 0 mM (*p* = 2.0 × 10^−2^) and 25 mM (*p* = 1.9 × 10^−2^) ColAlg gels, respectively (Figure 3D).

**Figure 4:**
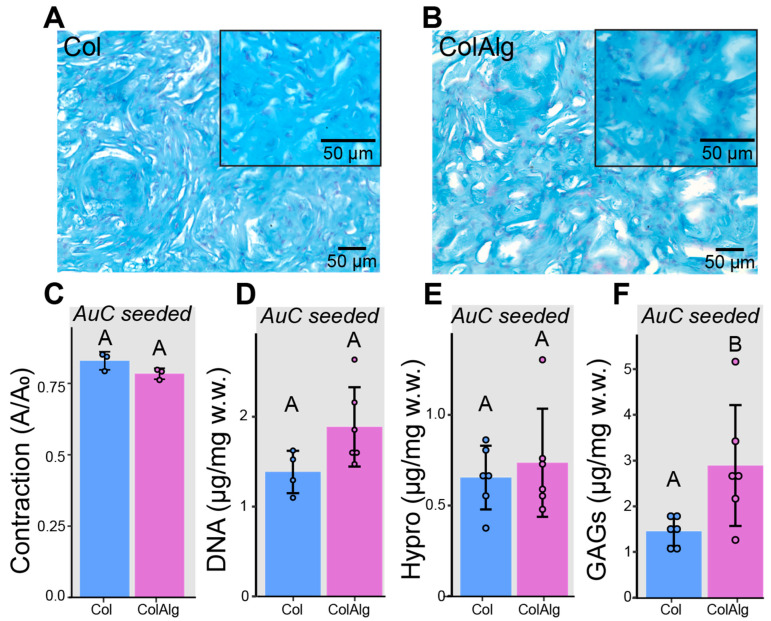
Alcian blue histology with fast red counterstaining of auricular chondrocyte-seeded Col (**A**) and ColAlg (**B**) constructs. Quantification of construct contraction (**C**) by area ratio, DNA (**D**), hydroxyproline (**E**), and glycosaminoglycans (**F**) according to sample wet weight. *n* = 3–6. Shared letters denote no significant difference.

**Figure 5:**
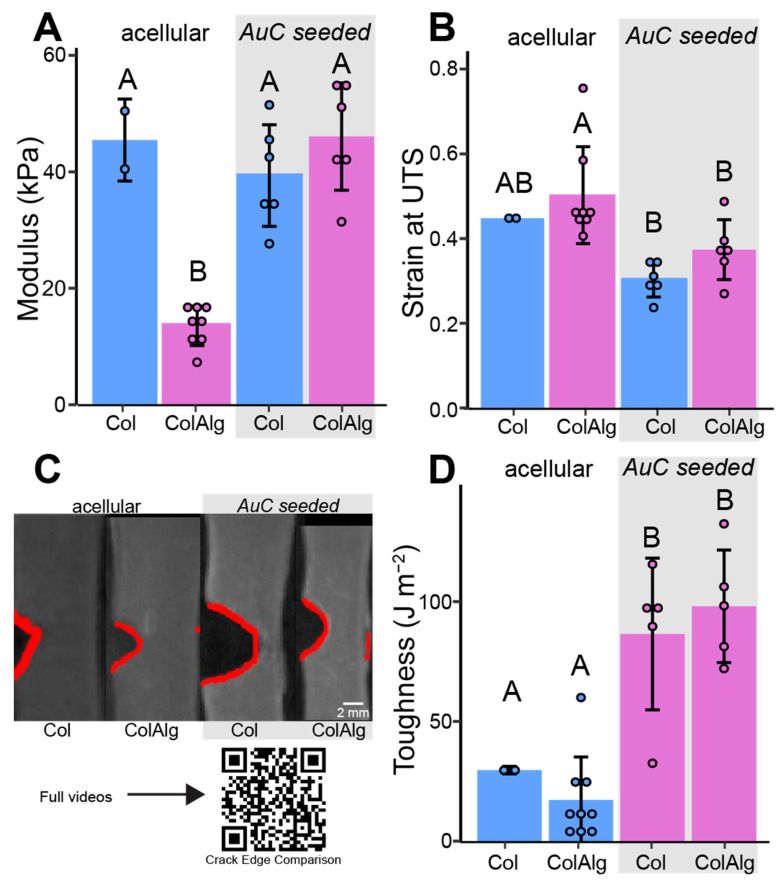
Calculated modulus (**A**) and strain at ultimate tensile stress (**B**) of acellular and cell-seeded constructs. Representative crack edge images (**C**) with automated crack tracking outlined in red. Calculated fracture toughness of acellular and cell-seeded constructs (**D**). *n* = 2–8. Shared letters denote no significant difference.

In addition to the mechanical characterization of acellular Col and ColAlg, gels were seeded with auricular chondrocytes and cultured in vitro for 2 weeks to investigate potential differences in cellular development. Alcian blue/fast red staining of histology sections was used to investigate glycosaminoglycan (GAG) deposition and cell distribution, while bulk biochemical analysis of GAGs, hydroxyproline (Hypro), and DNA was used to quantify extracellular matrix deposition and cell content, respectively. Similar GAG deposition and cell distribution was found in both Col and ColAlg, with robust chondrocyte development after 2 weeks of in vitro culture (Figure 4A,B). As anticipated, there were no accompanying differences in contraction (Figure 5C) and DNA content (Figure 5D). Likewise, the total content of hydroxyproline (which assesses collagen content) was not found to differ between Col and ColAlg (Figure 5E). Notably, after 2 weeks, a two-fold increase in GAG content was observed in ColAlg gels compared to Col (*p* = 2.7 × 10^−2^) (Figure 5F). In line with the similar deposition of GAGs and Hypro between Col and ColAlg, the viability of chondrocytes immediately after seeding in each gel was greater than 95%, with no difference between groups (Supplementary Figure S2).

The largely similar cellular development of Col and ColAlg gels was then compared to their mechanical development to determine differences in the mechanical integrity of the deposited extracellular matrix. After seeding with auricular chondrocytes, the modulus of Col was unchanged but increased in ColAlg by 3.4-fold (Figure 4A), resulting in an overall 2.1-fold increase in modulus with seeding (*p* = 1.8 × 10^−4^). Likewise, auricular chondrocyte seeding resulted in a slight (30 percent) decrease in the extensibility of ColAlg (*p* = 4.5 × 10^−2^), while extensibility was unchanged in Col (Figure 4B), resulting in a decrease in the extensibility of 0.15 ± 0.04 with seeding (*p* = 4.3 × 10^−4^). When grouped by material, however, ColAlg was found to be more extensible than Col (0.45 ± 0.12 vs. 0.34 ± 0.08, *p* = 2.8 × 10^−2^). Fracture toughness was similar for Col and ColAlg gels prior to auricular chondrocyte seeding. Following 2-week in vitro culture, the fracture toughness of both Col and ColAlg increased, by 2.9-fold in Col (*p* = 3.6 × 10^−2^) and 5.6-fold in ColAlg (*p* = 3.5 × 10^−5^).

## 4. Discussion

This study aimed to improve the toughness and extensibility of collagen hydrogels through the functionalization of collagen with alginate oligomers to enable metal–ion complexation. The conjugation of alginate to collagen, as confirmed with FTIR spectroscopy, had a minimal effect on collagen fibril nanostructure, gelation, and helical confirmation as confirmed via SEM, oscillatory shear rheology, and circular dichroism spectroscopy, respectively. Conjugation also enabled the calcium-dependent tuning of collagen’s tensile properties through the formation of alginate–ion complexes. This complexation resulted in the improved stiffness, extensibility, and toughness of modified gels overall. Notably, modified hydrogels still supported robust tissue formation by auricular chondrocytes seeded in gels. Specifically, DNA and Hypro were similar between groups, with two-fold higher GAG in ColAlg after 2 weeks in culture. Seeding with auricular chondrocytes improved modulus in ColAlg two-fold, while extensibility was largely maintained, potentially due to the observed 5.6-fold increase in fracture toughness in ColAlg following culture. Modulus and extensibility were meanwhile unchanged following culture for unmodified Col.

Several groups have used covalent crosslinking strategies in collagen to improve gel mechanics, through free-radical photochemistry [49], various enzymatic routes, [50,51,52] and non-enzymatic chemistry [53,54,55]. While these strategies have been somewhat effective in improving the moduli and strength of the resultant materials, they generally result in decreased extensibility and disrupt native structural and functional properties, [56,57,58] namely the formation of a uniform fibrillar network structure and thermal gelation. This disruption can be attributed to the interruption of collagen–collagen interactions in the main helical and telopeptide regions of collagen, which drive the formation of the characteristic helical structure [59] and fibrillogenesis, [39,60,61] respectively. The functionalization of collagen at the Nterminal peptide was therefore a promising target for strategies that maintain essential collagen–collagen interactions in the helical and telopeptide domains.

N-terminal conjugation has been demonstrated with high specificity in a variety of proteins through reductive amination. This reaction yields conjugation through linking the aldehyde group of a ligand to the N-terminal amine of a protein, as catalyzed through a reducing agent such as sodium cyanoborohydride. Fourier-transform IR spectroscopy is an effective means of investigating the attachment of un-tagged molecules to collagen. However, the method of comparing the integration area of the modification peak to stable collagen amide I or II peaks is made difficult in this case due to the relatively small size of the modification (~3 kDa) compared to the size of a collagen molecule (115 kDa), meaning that the changes in peak area ratio were expected to be small. Additionally, the use of a 5 cm^−1^ step size precludes the deconvolution of the amide I peak in our spectra, which may otherwise provide insight into potential conformational changes due to conjugation. Nonetheless, the comparison of ColAlg area ratio to a calibration curve of appropriate collagen–alginate mixtures resulted in a calculated reaction extent of 0.73 ± 0.29. While collagen has been identified as a key extracellular matrix analog for various cell culture systems, its use is often limited by a lack of versatility due to difficulty in making structural or functional modifications without disrupting native fibril structure or gelation. Thus, the N-terminal conjugation strategy employed here, while effective in modulating the extensibility and toughness of collagen as shown in this work, may also be effective in other functionalization strategies such as biomarker tagging, covalent crosslinking, or surface binding.

Due to the alginate functionalization of collagen achieved in this work, the modulus and work density increased with calcium concentration, with a peak in work density at 25 mM. This increase in modulus with calcium concentration is likely due to complexation of alginate oligomers with calcium ions in solution, similar to prior work with alginate–polyacrylamide hydrogels [26]. Conversely, modulus decreased with calcium concentration in control collagen, with a commensurate decrease in work density. Though somewhat counterintuitive due to complexation of glutamic acid residues with calcium ions in the resultant gels, [62] this finding is well supported in the context of denaturation and competitive inhibition. Due to the complexation of glutamic acid with calcium in solution (prior to gelation), both helical stability and subsequent fibril formation are decreased [63]. This behavior is also consistent with the behavior or collagen in ionic solutions more generally, where increased ionic strength decreases network strength [64]. In the context of alginate functionalization in this work, however, this calcium–glutamate binding is in competition with calcium–alginate complexation, leading to the competing effects observed.

Despite the striking effect of conjugation on the calcium-dependent mechanics of functionalized ColAlg gels, there was no accompanying disruption to the native fibril, network, or helical structure. A such, the cytocompatibility of ColAlg gels was expected to match that of unmodified Col. Indeed, the morphology of the auricular chondrocytes seeded on each gel after two weeks was similar, as were the quantifications of DNA and collagen content. Notably, GAGs were significantly higher in ColAlg. Increased GAG deposition in lower-modulus hydrogels has been well established in articular chondrocytes. It is thus not surprising that auricular chondrocytes respond to differences in gel modulus similarly, depositing a GAG matrix to achieve their ‘set point’ modulus. Indeed, the modulus of ColAlg constructs increased significantly after culture, matching that of Col after two weeks.

While the effect of auricular chondrocyte culture on extensibility was only marginal, increases in modulus were only found in ColAlg, and not Col. Fracture toughness was greatly increased for both materials after culture. With a five-fold overall increase in fracture toughness following in vitro culture, the cellular and extracellular matrix content developed over two weeks constitutes a significant improvement in material performance not found in either the modulus or extensibility data. This increase in the energy needed to propagate a crack through the material represents a step forward in the design of tissue-engineered cartilage materials, which are generally lower in both modulus and toughness. Additionally, this finding motivates the use of fracture toughness analysis of tissue-engineered materials, as traditional bulk mechanical testing may not fully encompass the effects of cellular development on those biomaterials. This is especially relevant given that the complete removal of defect sites in the context of cellular materials is not feasible, making fracture toughness a more reliable measure of failure properties than work density or strain at failure [42].

Current state-of-the-art materials such as costal cartilage or porous polyethylene (MedPor^®^), despite matching (or indeed exceeding) the modulus of native auricular cartilage, are significantly more brittle, contributing to the incidence of implant failure. In the context of pediatric microtia treatment, implant materials must be both flexible and durable to withstand frequent bending and extension of the exposed ear. Indeed, the use of plastic cage materials surrounding collagen gel implants has been demonstrated as an effective strategy for improving the durability and shape retention of auricular constructs [65]. However, these cage materials have not been evaluated on the basis of toughness or extensibility and are still expected to be brittle in comparison to native auricular cartilage. Indeed, the characterization of materials for auricular cartilage repair has generally been limited to linear mechanical properties, which do not encompass the high strains experienced by constructs in vivo.

This study investigated the effect of alginate functionalization on the cytocompatibility and cellular development of collagen hydrogels for two weeks in culture. However, longer-term cultures of seeded gels at multiple calcium concentrations would be needed to investigate the full effects of material and calcium–alginate complexation on the in vitro development of auricular cartilage constructs. In this relatively short-term culture, the modulus, work density, and toughness achieved were low compared to native auricular cartilage (with a modulus of 10–20 MPa [66,67]). Additionally, the cyclic fatigue loading of gels was not performed to elucidate the failure properties of each material. Despite these lower mechanical properties, it is worth noting that the most important considerations for auricular cartilage repair are shape retention and integration with the adjacent perichondrium and dermis, both of which contribute significantly to overall auricular mechanics [66]. As such, lower moduli and toughness at early time points may be necessary for tissue-engineered cartilage so that cellular development and integration can be readily achieved. This is contrasted with tissues such as articular cartilage or meniscal fibrocartilage, where in vivo loading necessitates moduli and strength of at least the same order of magnitude as native tissue to prevent failure.

Overall, this study demonstrated the potential for the N-terminal functionalization of collagen to achieve calcium-dependent increases in extensibility and toughness. Alginate-functionalized collagen maintained robust fibrillogenesis and helical structure, and thermal gelation resulted in gels with storage moduli on par with unmodified collagen gels. Further, alginate conjugation enabled calcium-dependent increases in modulus, toughness, and extensibility compared to collagen alone. Given the maintenance of native collagen fibril and helical structure, the cytocompatibility of alginate-functionalized collagen was likewise on par with unmodified collagen, with extracellular matrix deposition the same or higher in ColAlg compared to Col. While no differences were found between the mechanical properties of ColAlg and Col following auricular chondrocyte seeding, this study highlights the utility of extensibility and fracture toughness as key benchmarks for the cellular development of biomaterials generally. In tissue engineering applications with high material deformations in vivo, both the alginate-functionalized collagen as well as the fracture analysis testing methodology outlined in this study may be especially relevant.

## Data Availability

Data available on request.

## Supplementary Materials

The following supporting information can be downloaded at: https://www.mdpi.com/article/10.3390/bioengineering10091037/s1, Figure S1: Molecular weight of alginate oligomers; Figure S2: Live/dead staining of auricular chondrocyte-seeded gels; File S1: Crack Edge Analysis_Bioengineering.
