# Supplementary material for: Alginate Conjugation Increases Toughness in Auricular Chondrocyte Seeded Collagen Hydrogels
Source: Bioengineering (Basel). 2023 Sep 4;10(9):1037. doi: 10.3390/bioengineering10091037 (PMC10526064; doi:10.3390/bioengineering10091037)
Supplement: Supplementary file 1 [file bioengineering-10-01037-s001.zip › Bioengineering_Supplementary Material_R1.pdf]

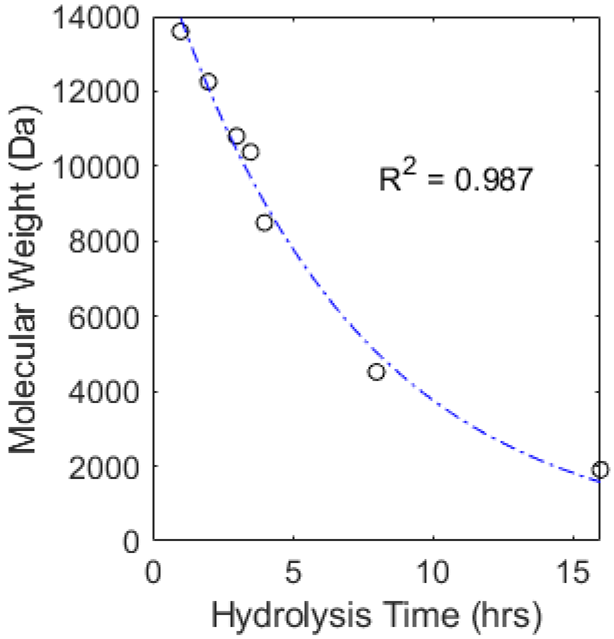

Figure S1. Molecular weight of alginate oligomers vs. hydrolysis time at pH 3.5 under nitrogen at 95°C. n = 3 per hydrolysis time condition.

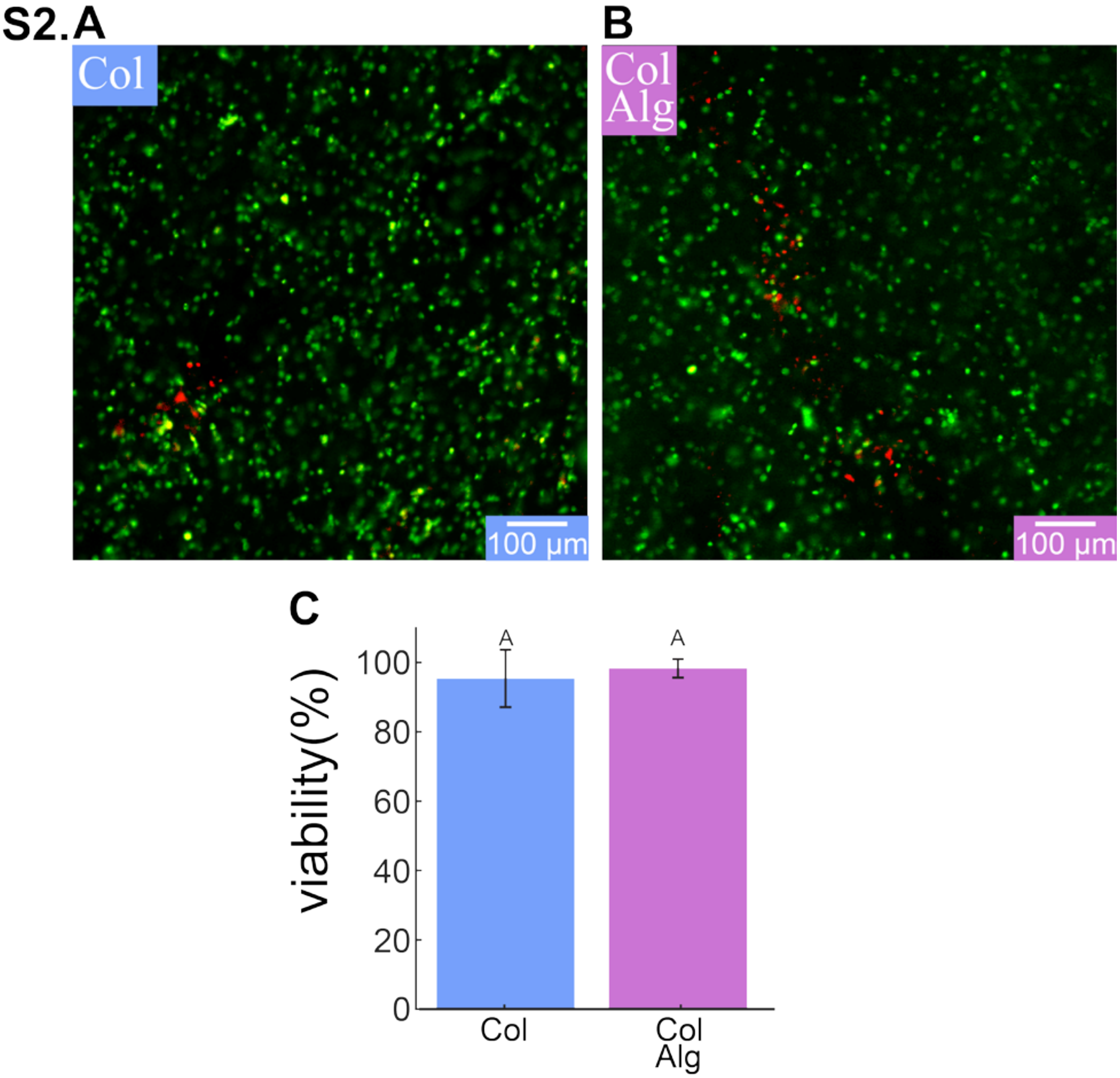

Figure S2. Live/dead staining of auricular chondrocyte-seeded Col **(A)** and ColAlg **(B)** gels, with calculated viability of each gel **(C)**. n = 10. Shared letters denote no significant difference.
